# Large Language Models vs. Professional Resources for Post-Treatment Quality-of-Life Questions in Head and Neck Cancer: A Cross-Sectional Comparison

**DOI:** 10.3390/curroncol32120668

**Published:** 2025-11-28

**Authors:** Ali Alabdalhussein, Mohammed Hasan Al-Khafaji, Shazaan Nadeem, Maham Basharat, Hasan Aldallal, Mohammed Elkrim S. Mohammed, Sahar Alghnaimawi, Ali Al Yousif, Juman Baban, Soroor Hamad, Ibrahim Saleem, Sarah Mozan, Manish Mair

**Affiliations:** 1 Department of Otolaryngology, University Hospitals of Leicester, Leicester LE1 5WW, UK; mhkhachi@doctors.org.uk (M.H.A.-K.); shazaan.nadeem@nhs.net (S.N.); mohammed.mohammed21@nhs.net (M.E.S.M.); 2 Department of General Surgery, Northern Health and Social Care Trust, Coleraine BT52 1HS, UK; maham.basharat@northerntrust.hscin.net; 3 Department of Trauma and Orthopaedics, University Hospital of Derby and Burton NHS Foundation Trust, Derby DE22 3NE, UK; hasan.aldallal@nhs.net; 4 ENT Head and Neck Department, Russell’s Hall Hospital, Dudley Group NHS Foundation Trust, Dudley DY1 2HQ, UK; sahar.al-ghnaimawi@nhs.net; 5 Independent Researcher, London SA61 2EL, UK; ali.alyousif@doctors.org.uk; 6 Care of the Elderly Department, Chelsea and Westminster NHS Trust London, London TW7 6AF, UK; juman.baban@nhs.net; 7 Neonatal Unit, South Tees Hospital, James Cook University Hospital Marton Road, Middlesbrough TS4 3BW, UK; soroor.hamad@nhs.net; 8 Emergency Assessment Unit, Salford Royal NHS Foundation Trust, Manchester M6 8HD, UK; ibrahim.saleem@srft.nhs.uk; 9 Paediatrics Department, Croydon University Hospital, Croydon City CR7 7YE, UK; sarah.mozan@nhs.com

**Keywords:** head and neck cancer, quality of life, large language models, artificial intelligence, patient education, Macmillan Cancer Support, CURE Today, readability assessment, patient information resources

## Abstract

Background: Recently, patients have been using large language models (LLMs) such as ChatGPT, Gemini, and Claude to address their concerns. However, it remains unclear whether the readability, understandability, actionability, and empathy meet the standard guidelines. In this study, we aim to address these concerns and compare the outcomes of the LLMS to those of professional resources. Methods: We conducted a comparative cross-sectional study by following the relevant items of the Strengthening the Reporting of Observational Studies in Epidemiology (STROBE) checklist for cross-sectional studies and using 14 patient-style questions. These questions were collected from the professional platforms to represent each domain. We derived the 14 domains from validated quality-of-life instruments (EORTC QLQ-H&N35, UW-QOL, and FACT-H&N). Fourteen Responses were obtained from three LLMs (ChatGPT-4o, Gemini 2.5 Pro, and Claude Sonnet 4) and two professional sources (Macmillan Cancer Support and CURE Today). All responses were evaluated using the Patient Education Materials Assessment Tool (PEMAT), DISCERN instrument, and the Empathic Communication Coding System (ECCS). Readability was assessed using the Flesch Reading Ease and Flesch-Kincaid Grade Level metrics. Statistical analysis included one-way ANOVA and Tukey’s HSD test for group comparisons. Results: No differences were found in quality (DISCERN), understandability, actionability (PEMAT), and empathy (ECCS) between LLMS and professional resources. However, professional resources outperform the LLMs in readability. Conclusions: In our study, we found that LLMs (ChatGPT, Gemini, Claude) can produce patient information that is comparable to professional resources in terms of quality, understandability, actionability, and empathy. However, readability remains a key limitation, as LLM-generated responses often require simplification to align with recommended health-literacy standards.

## Simple Summary

Patients with head and neck cancer often have many concerns and questions about their quality of life after treatment, such as speech, swallowing, or appearance. Traditionally, to address these questions, patients either see a doctor or search online in trusted professional resources. However, recently, people have started to use AI chatbot models to address these concerns, like ChatGPT, Gemini, and Claude, to find quick and personal advice online. It is unclear whether these tools are reliable and understandable. Our study aims to compare answers from these AI systems with those from established cancer organisations. We carefully assess how easy these answers are to read and whether they are clinically accurate. We also evaluate if AI chatbots can show empathy in their responses.

## 1. Introduction

Head and neck cancer is among the most common cancers worldwide, ranking as the seventh most prevalent type. Its incidence and prevalence have been increasing in recent decades, with over 660,000 new cases and approximately 325,000 deaths each year globally [1,2]. With the advancement of the treatment, patients still experience a significant impact on their quality of life, such as swallowing difficulties, speech impairment, altered appearance, fatigue, pain, and psychological distress. With such problems, patients would have multiple concerns, and helping them find accurate, precise, and supportive information would be critical and a cornerstone in their recovery journey. Not only trustworthiness but also the readability of information is vital. Readability is particularly important for oncology patients, who often face complex and emotionally challenging medical information during diagnosis and treatment; using clear and accessible language helps support understanding, adherence, and emotional reassurance. As a very first attempt to address this, we have used a hard-copy patient leaflet that provided limited information and did not meet patients’ requirements. As a result, official medical organisations, such as the NHS, began to develop online platforms, such as NHS Conditions, in the early 2000s [3,4]. Transitioning from printed patient leaflets to an online platform with extensive information has been a lengthy journey. It is very popular nowadays to seek health information from online resources such as the NHS and Macmillan websites [5]. The evolution from printed leaflets to online patient information platforms has significantly improved accessibility and content breadth. Building upon these advancements, the emergence of large language models (LLMs) such as ChatGPT, Gemini, and Claude represents the next stage in this progression introducing interactive, conversational tools capable of delivering personalized responses to patient queries. Patients have begun to utilise these chatbots to seek medical information and clinical advice, particularly on quality-of-life concerns, following medical or surgical treatment for head and neck cancer [6]. The accuracy and reliability of large language models are still a topic of debate. Previous studies have compared the accuracy and reliability of AI-generated medical information with physician-provided advice, demonstrating variable performance across different medical domains [7,8]. In contrast, other research has focused on the readability and accessibility of patient education materials, highlighting persistent challenges in ensuring that health information is understandable to diverse patient populations [9].

Although several recent studies have evaluated how large language models (LLMs) such as ChatGPT generate medical information, these have largely focused on general disease explanations or single-system conditions. Most of these studies did not evaluate outputs against validated educational standards. Al Sammarraie et al. in their paper, “The Use of Large Language Models in Generating Patient Education Materials: A Scoping Review,” after reviewing 69 studies, found that accuracy and readability of responses were the main metrics of evaluation. However, only a few studies employed formal assessment frameworks [10]. Therefore, the communicative and empathic dimensions of AI-generated responses remain underexplored, particularly for complex cancers that profoundly affect daily functioning and self-image, such as head and neck malignancies. On the other hand, Current professional resources, such as those from organisations like Macmillan Cancer Support and Cancer Research UK, provide carefully reviewed advice. Similarly, clinicians working in ENT, maxillofacial, and oncology settings are trained to handle these issues with nuance and clinical judgment. This study aims to evaluate the quality of responses generated by large language models (LLMs) to common quality-of-life (QoL) questions expressed by patients with head and neck cancer. It compares these AI-generated answers with professional resources from Macmillan and Cure, the website’s publicly available patient-facing resources produced by NHS-affiliated organisations, and expert-written responses, where available. The aim is to assess how effectively current LLMs meet both the informational and communicative needs of this patient population.

To our knowledge, no previous research has systematically compared LLMs with professional patient-education resources using validated tools for readability, understandability, actionability, and empathy. By grounding our questions in three established quality-of-life instruments (EORTC QLQ-H&N35, UW-QOL, and FACT-H&N), this study introduces a novel, evidence-based framework for evaluating whether modern LLMs can meet the informational and emotional needs of patients recovering from head and neck cancer. It also highlights the possible role of LLMs in patient education and emotional support throughout their recovery journey.

## 2. Materials and Methods

**Figure 1:**
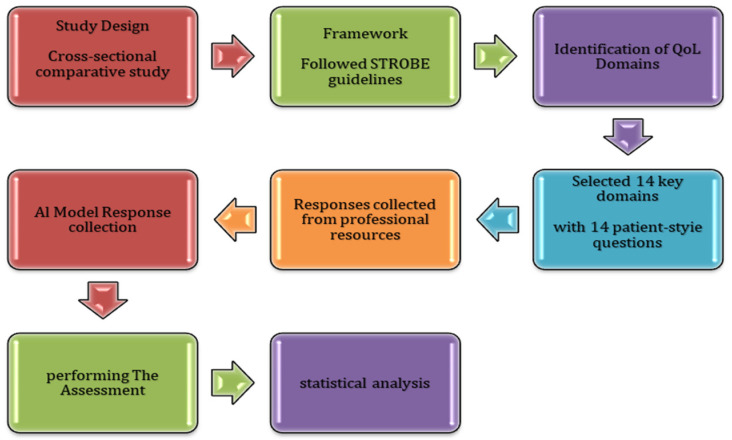
Methodological Flowchart of the Cross-Sectional Comparative Study Design.

We conducted this cross-sectional comparative study in accordance with the STROBE (Strengthening the Reporting of Observational Studies in Epidemiology) guidelines in July 2025 [11] (see STROBE checklist in the Supplementary Material File S1) Figure 1. To identify the most relevant quality of life concerns following treatment for head and neck cancer, we reviewed three widely validated patient-reported outcome instruments: the European Organisation for Research and Treatment of Cancer Quality of Life Questionnaire-Head and Neck 35 (EORTC QLQ-H&N35; EORTC Quality of Life Group, Brussels, Belgium) [12], the University of Washington Quality of Life questionnaire (UW-QOL; Department of Otolaryngology–Head and Neck Surgery, University of Washington, Seattle, WA, USA) [13], and the Functional Assessment of Cancer Therapy-Head and Neck questionnaire (FACT-H&N; FACIT Measurement System, Evanston, IL, USA) [14]. From these instruments, we selected 14 domains that represent key areas of concern for patients. Fourteen representative patient questions were derived directly from the survivorship and quality-of-life sections of professional head and neck cancer resources, primarily the Macmillan Cancer Support website [15] (After treatment for head and neck cancer, Living with and beyond cancer) and the CURE Today patient stories and survivorship pages [16]. Each domain corresponded to one of the major areas addressed by validated QoL instruments (e.g., EORTC QLQ-H&N35, UW-QOL).

When multiple phrasings of a question existed (e.g., ‘How can I eat after radiotherapy?’ vs. ‘Will I be able to swallow normally?’), the version most representative of patient-facing language and appearing first within the relevant webpage was selected. No editing, abbreviation, or paraphrasing was performed. Each extracted question was used verbatim when prompting the large language models.

These platforms are widely recognised for providing reliable, accessible content on cancer in general and head and neck cancer specifically. Professional resources were selected based on the following criteria: (1) freely accessible online without login restrictions; (2) dedicated to head and neck cancer survivorship content authored or reviewed by oncology professionals; (3) providing detailed, patient-facing information on quality-of-life issues after treatment. Based on these criteria, Macmillan Cancer Support (UK-based) and CURE Today (US-based) were included. CRUK and NHS resources were excluded due to limited coverage of post-treatment quality-of-life domains and greater focus on general cancer overviews. The complete list of questions is provided in Table 1. To compare the information available to patients, we obtained responses from the above-mentioned platforms. We extracted the relevant sections directly and compiled them into Word documents for analysis. We also evaluated three widely used large language models:

- ChatGPT-4o (OpenAI, released May 2024, accessed via chat.openai.com), temperature = 0.7, maximum tokens = 800, default system prompt (‘You are ChatGPT, a large language model trained by OpenAI’).
- Gemini 2.5 Pro (Google DeepMind, released July 2025, accessed via gemini.google.com accessed on July 2025), temperature = 0.8, default maximum output length (~1000 tokens).
- Claude Sonnet 4 (Anthropic, released May 2025, accessed via claude.ai), temperature = 0.7, default context window (~2000 tokens).

For each question, a new conversation thread was initiated to avoid contextual carryover. Each question was submitted once per model to replicate a typical single-user interaction rather than an averaged multi-run approach. ChatGPT has been widely adopted, with over 60% of users preferring it for medical information due to its convenience and comprehensiveness [17]. Gemini and its medical variant, Med-Gemini, demonstrate advanced biomedical reasoning and data synthesis capabilities [18]. Claude Sonnet 4 provided a broader comparison across distinct model architectures and conversational designs. We posed the same set of 14 patient-style questions to each model, preceded by the statement, “I have been diagnosed with head and neck cancer,” to ensure the responses were framed appropriately for the clinical context. To minimise algorithmic bias and better replicate a typical patient’s first-time experience, we used a guest (non-logged-in) session for ChatGPT to avoid any influence of prior data or personalisation. Since Gemini and Claude require user accounts, we created new accounts specifically for this study. We collected all responses and saved them in Word documents for subsequent evaluation.

We assessed the readability of all responses using the Flesch Reading Ease (FRE) and Flesch-Kincaid Grade Level (FKGL) metrics [19], employing the built-in readability tools available in Microsoft Word. Before calculating readability metrics, all responses were preprocessed to ensure consistency. Non-textual elements (e.g., URLs, bullet points, formatting symbols) were removed, and bullet lists were converted into complete sentences to maintain grammatical integrity. No manual edits or content alterations were made beyond these steps. We documented these results in a master Excel file for analysis. Before commencing the formal evaluation, two reviewers received standardised training on the application of the PEMAT, DISCERN, and ECCS tools using sample materials not included in the final dataset. A calibration exercise was conducted on sample responses until full conceptual agreement was achieved. Inter-rater reliability was assessed on 10% of responses using Cohen’s κ for categorical instruments (DISCERN, ECCS) and a two-way random effects intraclass correlation coefficient [ICC (2, 1)] for continuous measures (PEMAT). Agreement was substantial to excellent across metrics (κ = 0.79 for DISCERN; κ = 0.82 for ECCS; ICC = 0.86 for PEMAT-U; ICC = 0.88 for PEMAT-A).

When the two reviewers disagreed, they discussed their assessments to reach consensus; if necessary, a third reviewer consulted to resolve any outstanding discrepancies. After the training, Two independent reviewers, blinded to the source of the materials, evaluated all content using the Patient Education Materials Assessment Tool (PEMAT) [20] to assess understandability and actionability, the DISCERN instrument to appraise the quality and reliability of treatment information [21], and the Empathic Communication Coding System (ECCS) [22] to quantify the presence of empathic communication features. The Empathic Communication Coding System (ECCS), originally developed for analysing clinician–patient dialogue, was adapted for use with written text. Each AI or professional response was treated as a single narrative unit and evaluated across four domains: acknowledgement, validation, support, and information-coupled empathy, on a 1–5 scale, with higher scores indicating greater empathy. The adaptation focused on linguistic indicators of empathic intent rather than conversational turn-taking. Inter-rater reliability for the ECCS was substantial (κ = 0.82), confirming consistency in its textual application. We calculated descriptive statistics for each outcome measure. To compare mean scores across the four groups (professional resources, ChatGPT, Gemini, and Claude), we performed one-way analysis of variance (ANOVA) tests for each metric. Before conducting one-way ANOVA, data distributions for each outcome measure were tested for normality using the **Shapiro–Wilk test** and for homogeneity of variance using **Levene’s test**. All variables met these assumptions (*p* > 0.05). To account for multiple parallel comparisons across different evaluation tools, *p*-values were adjusted using the **Holm method** to control the family-wise error rate. When statistically significant differences were identified (*p* < 0.05), we conducted Tukey’s honest significant difference (HSD) tests to explore pairwise comparisons. We have utilised built-in features in Microsoft Excel to perform ANOVA tests and SPSS software (version 26) for all other statistical analyses.

## 3. Results

Four information sources, professional resources, GPT, Gemini, and Claude, were evaluated across six validated metrics: Flesch Reading Ease (FRE), Flesch-Kincaid Grade Level (FKGL), DISCERN, PEMAT-Understandability, PEMAT-Actionability, and the Empathic Communication Coding System (ECCS).

### 3.1. Readability

#### 3.1.1. Flesch Reading Ease (FRE)

**Figure 2:**
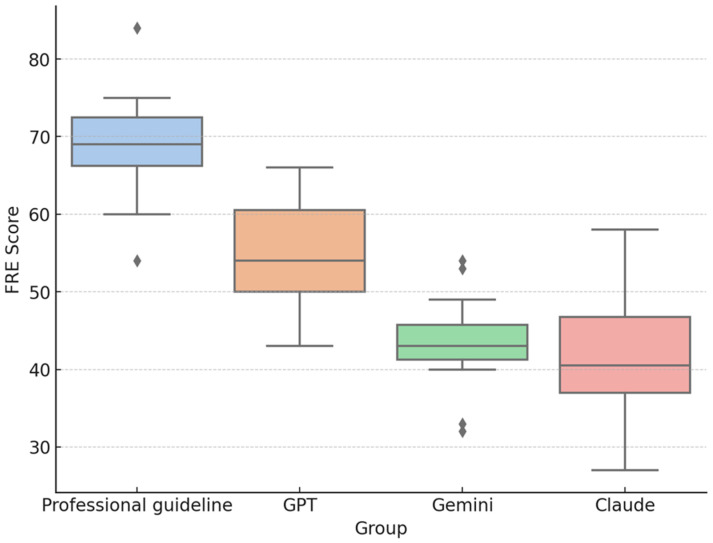
FRE distribution by source (Professional resources *n* = 14, ChatGPT *n* = 14, Gemini *n* = 14, Claude *n* = 14; total *N* = 56). Higher FRE values indicate easier readability.

Regarding readability, the FRE score was highest for professional resources (M = 69.1, SD = 7.0), followed by ChatGPT (M = 54.8, SD = 6.9), Gemini (M = 43.4, SD = 6.3), and Claude (M = 41.6, SD = 7.8). Figure 2 illustrates the distribution of Flesch Reading Ease (FRE) scores across sources. *Higher FRE values indicate easier readability, whereas lower scores represent more complex text.* A one-way ANOVA revealed a significant difference in FRE scores across the four sources, F(3, 52) = 45.5, *p* < 0.001, **η^2^ = 0.72**, **95% CI [0.61–0.79]**, indicating a large effect. Tukey’s HSD post hoc analysis showed that ChatGPT performed significantly better than Gemini and Claude (*p* < 0.001), while professional resources outperformed all AI models (*p* < 0.001). Claude and Gemini did not differ significantly (*p* = 0.907) (Table 2 and Table 3). In practical terms, professional resources were approximately 1.5–2.0 grade levels easier to read compared to LLM-generated responses, reflecting a notable gap in linguistic accessibility. Among the AI models, ChatGPT produced slightly simpler and more readable text than Gemini and Claude, suggesting modest differences in language structure and sentence complexity across models.

#### 3.1.2. Flesch-Kincaid Grade Level (FKGL)

**Figure 3:**
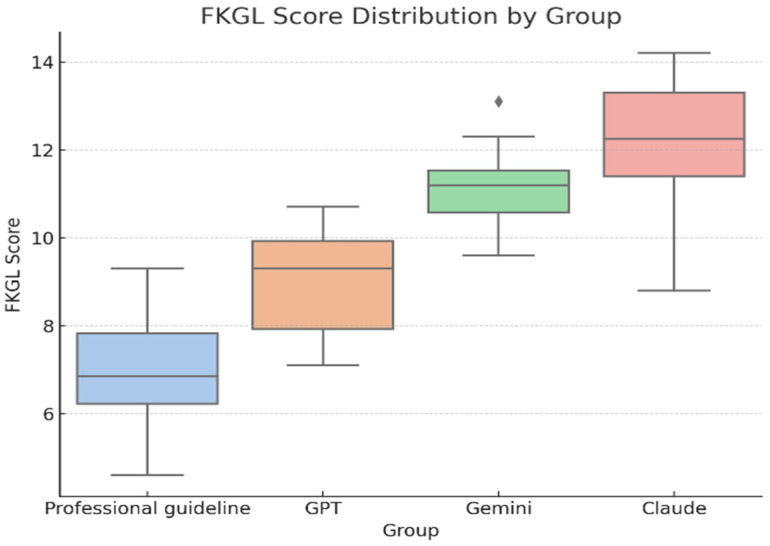
FKGL distribution by source (Professional resources *n* = 14, ChatGPT *n* = 14, Gemini *n* = 14, Claude *n* = 14; total *N* = 56). Higher FKGL values indicate greater reading difficulty.

To further reinforce the FRE results, we analysed the FKGL scores across all four sources (*n* = 56; 14 responses per group). The lowest reading grade level was observed for professional resources (M = 6.9, SD ≈ 1.4), followed by ChatGPT (M = 9.0, SD ≈ 1.5), Gemini (M = 11.1, SD ≈ 0.9), and Claude (M = 12.0, SD ≈ 2.0) Figure 3 illustrates the distribution of Flesch–Kincaid Grade Level (FKGL) scores across sources. Higher FKGL values indicate greater reading difficulty, whereas lower scores correspond to easier readability. A one-way ANOVA confirmed a statistically significant difference in FKGL scores among the groups, F(3, 52) = 49.6, *p* < 0.001, η^2^ = 0.74, 95% CI [0.63–0.80], indicating a large effect size. Claude produced significantly more complex content than ChatGPT and professional resources (*p* < 0.001), while ChatGPT remained significantly less readable than the professional reference (*p* = 0.0004). Claude and Gemini did not differ significantly (*p* = 0.096). Tukey’s HSD post hoc analysis further showed that ChatGPT performed considerably better than Gemini and Claude (*p* < 0.001), whereas professional resources outperformed all AI models (*p* < 0.001) (Table 4 and Table 5). In practical terms, professional resources were approximately 1.5–2.0 grade levels easier to read than LLM-generated outputs, reflecting simpler sentence structure and fewer technical terms. Among the AI models, ChatGPT produced content closer to patient-friendly readability compared to Gemini and Claude, which tended to use longer sentences and more complex phrasing.

### 3.2. Quality

**Figure 4:**
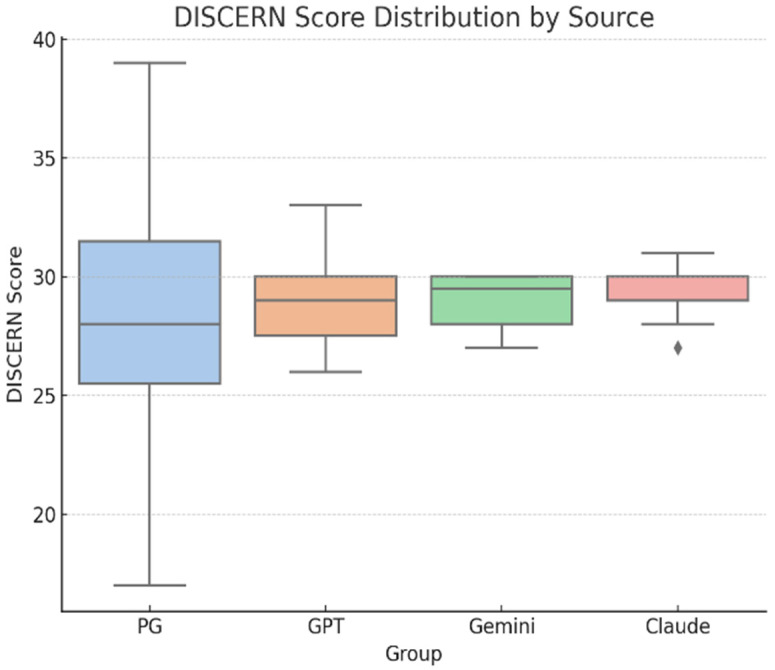
DISCERN distribution by source (Professional resources *n* = 14, ChatGPT *n* = 14, Gemini *n* = 14, Claude *n* = 14; total *N* = 56). Higher DISCERN values indicate better quality and reliability of health information.

For the quality of online information, we used DISCERN scores, which were similar across all sources (***n* = 56; 14 responses per group**): professional resources (M = 27.93, SD ≈ 5.1), ChatGPT (M = 29.00, SD ≈ 2.2), Gemini (M = 29.21, SD ≈ 1.7), and Claude (M = 29.07, SD ≈ 1.5) (Figure 4). A one-way ANOVA revealed no significant differences in DISCERN scores, F(3, 52) = 0.48, *p* = 0.70, **η^2^ = 0.03, 95% CI [0.00–0.10],** indicating a negligible effect size and suggesting that all sources performed comparably in terms of content quality (Table 6).

### 3.3. The Patient Education Materials Assessment Tool (PEMAT)

#### 3.3.1. PEMAT-Understandably

**Figure 5:**
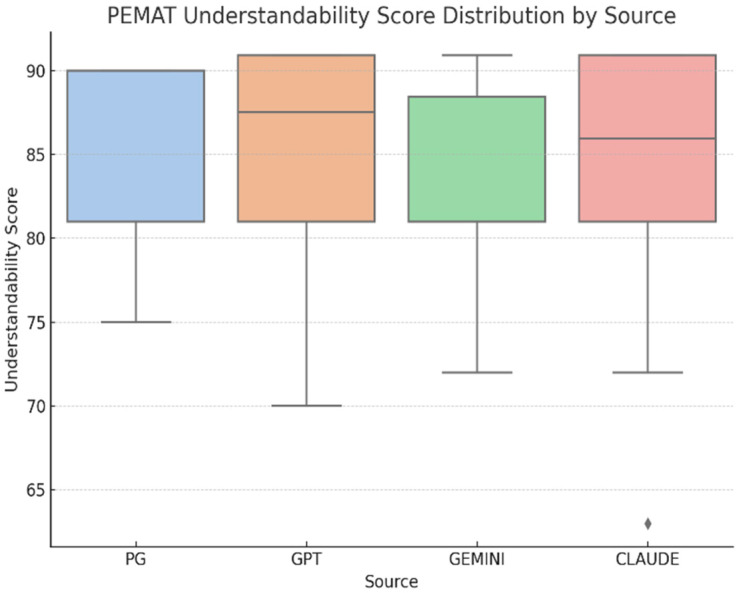
PEMAT-Understandability distribution by source (Professional resources *n* = 14, ChatGPT *n* = 14, Gemini *n* = 14, Claude *n* = 14; total *N* = 56). Higher PEMAT-Understandability scores indicate easier comprehension of information.

Regarding **PEMAT-Understandability**, scores were consistently high across all sources (***n* = 56; 14 responses per group**): professional resources (M = 85.29, SD ≈ 5.96), ChatGPT (M = 85.18, SD ≈ 5.89), Gemini (M = 81.89, SD ≈ 6.91), and Claude (M = 84.02, SD ≈ 6.85) (Figure 5). A one-way ANOVA indicated no significant differences in understandability among the groups, F (3, 52) = 0.68, *p* = 0.57, **η^2^ = 0.04, 95% CI [0.00–0.12]**, showing a negligible effect size and suggesting that all sources provided similarly understandable information (Table 7).

#### 3.3.2. PEMAT-Actionability

**Figure 6:**
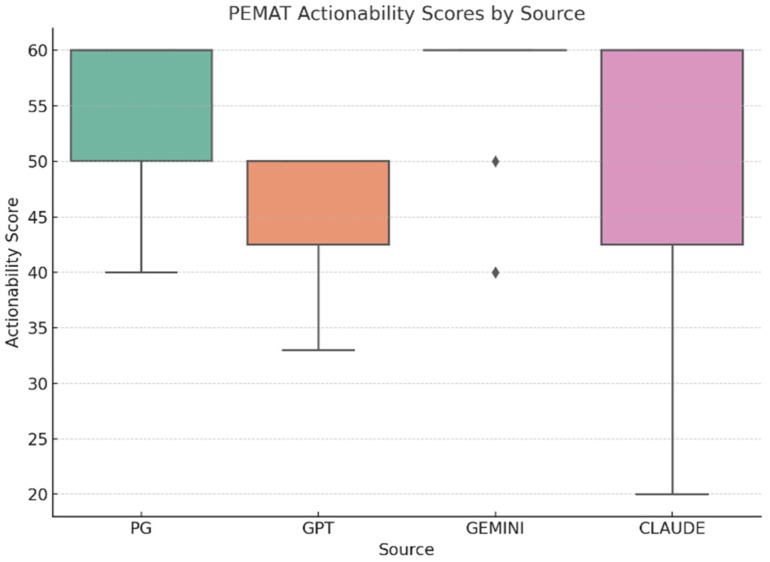
PEMAT-Actionability distribution by source (Professional resources *n* = 14, ChatGPT *n* = 14, Gemini *n* = 14, Claude *n* = 14; total *N* = 56). Higher PEMAT-Actionability scores indicate clearer guidance for patients on what actions to take.

**PEMAT-Actionability** scores were moderate and consistent across sources (***n* = 56**; **14 responses per group**): professional resources and Claude (M = 50.71), ChatGPT (M = 46.64), and Gemini (M = 51.43) (Figure 6). A one-way ANOVA found no statistically significant difference between groups, F (3, 52) = 0.72, *p* = 0.54, **η^2^ = 0.04**, **95% CI [0.00–0.13]**, indicating a negligible effect size and confirming similar performance across all sources in facilitating patient action (Table 8).

### 3.4. Empathic Communication Coding System (ECCS)

**Figure 7:**
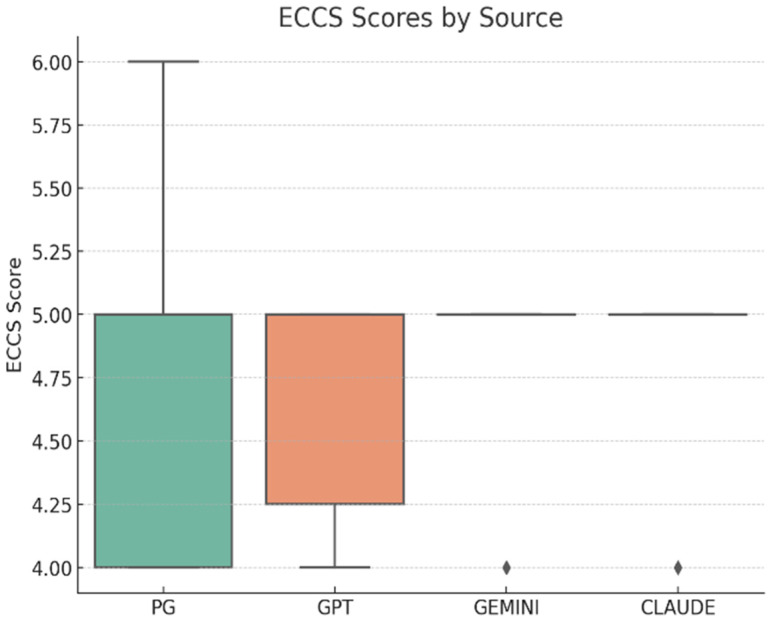
ECCS distribution by sources (Professional resources *n* = 14, ChatGPT *n* = 14, Gemini *n* = *14,* Claude *n* = 14; total *N* = 56). Higher scores indicate more empathic response.

Empathy was assessed using the Empathic Communication Coding System (ECCS), with average scores closely aligned across sources (***n* = 56; 14 responses per group**): professional resources (M = 4.64, SD ≈ 0.63), ChatGPT (M = 4.71, SD ≈ 0.47), Gemini (M = 4.79, SD ≈ 0.43), and Claude (M = 4.79, SD ≈ 0.43) (Figure 7). A one-way ANOVA revealed no significant differences in empathic communication among the groups, F(3, 52) = 0.27, *p* = 0.85, **η^2^ = 0.02, 95% CI [0.00–0.09]**, indicating a negligible effect size and confirming that all sources conveyed empathy at comparable levels (Table 9).

### 3.5. Summary

**Figure 8:**
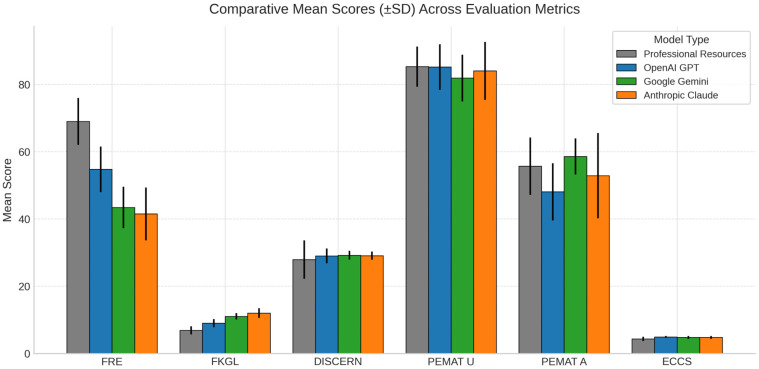
Comparison of mean scores and standard deviations across all metrics (Professional resources *n* = 14, ChatGPT *n* = 14, Gemini *n* = 14, Claude *n* = 14; total *N* = 56). Higher scores represent better readability, quality, understandability, actionability, or empathy, depending on the respective metric.

To summarise all the results, Figure 8 illustrates the comparative performance of professional resources (PG), ChatGPT, Gemini, and Claude across key evaluation metrics. PG demonstrated the highest Flesch Reading Ease (FRE) score (69.0), indicating the most readable content, while Claude had the lowest (41.5). Conversely, FKGL scores were lowest for PG (6.89) and highest for Claude (12.01), reflecting more complex language in the LLM outputs. DISCERN scores were broadly similar across all sources, with Gemini marginally leading (29.21). For understandability (PEMAT-U), PG and GPT scored similarly high (85.29 and 85.18, respectively), while Gemini led on actionability (PEMAT-A) with a mean of 58.57. ChatGPT achieved the highest empathic communication score (ECCS 4.93), closely followed by Gemini and Claude (4.79). Standard deviations varied more widely for actionability and FRE, particularly in Claude.

## 4. Discussion

### 4.1. Key Findings in Context

Our study found that professional resources outperform large language models (LLMs) in terms of reading feasibility; however, no difference was observed in quality, understandability, actionability, or empathy. These findings open two areas for discussion. First, a high readability level is a personaliseddvantage. In the UK, the National Health Service (NHS) estimates that over 40% of adults have difficulty understanding publicly available health information, and this figure rises above 60% when numerical data or statistics are included. Much of this difficulty stems from health materials being written at a literacy level higher than that of the general population, which limits their accessibility and effectiveness [23]. The National Centre for Education and the US Department of Education indicate that 36% of adults have only “basic” or “below basic” health literacy, with 43% of adults either at or below basic reading levels [24,25]. To address this, professional resources suggest that the reading level should be no greater than a sixth grade reading level [26,27]. Despite these recommendations, several studies have indicated that current patient education materials may be at a reading level that is too complex for most patients to comprehend [28,29]. Without an explicit request to reduce readability, complex readability in LLMs is expected [30,31,32]. Low readability scores could be due to several factors. First, we did not specify the reading level, which led to LLMs using the default setting and generating responses at an academic level when they related to medical knowledge, requiring a specific order to decrease the reading grade, such as ‘Rewrite the following at a 6th-grade reading level’ [33]. Second, the terminology used in these models is derived from the biomedical literature (e.g., PubMed, guidelines) [34]. Third, LLMs are trained on large text corpora, including academic writing. This can lead to output with complex, nested sentence structures, which reduce Flesch Reading Ease scores [35]. Additionally, long sentences, passive phrasing, and the use of complex medical terms make the text harder to read and lower its readability score.

On the other hand, no differences were observed in quality, understandability, actionability, and empathy, which is very promising. This suggests that LLMs can produce high-quality and trustworthy content.

With increased pressure on the health system worldwide, LLMs can offer invaluable service by supporting patients with head and neck cancer throughout their journey. Recent studies have found that LLMs are capable of producing high-quality medical information and can efficiently address patients’ concerns [36,37,38]. However, without improving the readability, LLMs would not be a recommended source of information, and the utility of LLMs may be limited in populations with low health literacy or among individuals for whom English is not a first language.

Beyond these quantitative findings, our results highlight the actual benefit of LLMs in practical settings generally and patient communication specifically. With direct supervision, these tools could enhance patient education by providing trustworthy information and being empathetic with more personalised responses. In oncology and head and neck cancer care, chatbots could support pre-treatment counselling, symptom education, and postoperative recovery guidance. However, their clinical integration must include safeguards against misinformation, clear accountability, and validation frameworks to ensure that AI complements, rather than replaces, clinician-patient interaction

Chatbots such as ChatGPT, Gemini, and Claude operate through algorithms that may introduce data or algorithmic bias, potentially resulting in inaccurate or inequitable recommendations. Ensuring transparency, explainability, and responsible data handling is essential to preserve trust and safeguard patient privacy [39].

AI chatbots hold great potential to transform patient care and healthcare delivery; however, their responsible and ethical use depends on addressing complex ethical considerations. By prioritising patient welfare, transparency, fairness, and collaboration. It is a responsibility of the healthcare systems, technologists, and clinicians to ensure that these technologies advance healthcare while maintaining the highest ethical standards. In summary, while large language models have a high readability level, their outstanding performance in understandability, empathy, and informational quality is encouraging. Despite the high understandability, readability remained low. While these are distinct constructs, they are closely related in shaping how patients engage with written information. This finding may be explained by the logical structure, coherent flow, and familiar context of the content, which aided comprehension despite complex wording. Additionally, LLMs tend to generate longer sentences and use technical or formal vocabulary, which can reduce readability scores even when the overall message remains clear and easy to follow [40]. As these models can be easily trained, we suggest integrating AI with professional resources, where a well-controlled chatbot can produce high-quality, low-readability information. This can be further optimized through prompt engineering for example, instructing models to generate text at a sixth grade reading level and by applying plain language principles to ensure clarity and accessibility for all patients.

### 4.2. Limitations

This study has several limitations. First, our evaluation was based on a single static question generated for each domain. In real life, patients are more likely to engage in several prompts with an AI chatbot.

Second, our assessment was conducted entirely in the English language, which means it may not be suitable for patients who speak other languages. This introduces potential language bias, as readability and comprehension levels can vary across linguistic and cultural contexts, potentially limiting the generalisability of our findings to non-English-speaking populations.

Third, we carried out this assessment at a single point in time (July 2025). Since these models are updated frequently, and any upcoming update might change their responses

We also did not involve patients directly in the assessment process. While we used validated tools to measure understandability, empathy, and information quality, we cannot say for sure how real patients would experience these materials, whether they would feel reassured, confused, or empowered. Furthermore, the DISCERN tool evaluates the reliability and evidence quality of health information, it does not directly confirm factual or clinical accuracy; therefore, future studies should include expert validation to assess potential misinformation risks.

A simple ANOVA approach was sufficient to address the study’s comparative objectives; future research may incorporate inter-rater agreement measures and effect size analyses to provide additional statistical depth and validation.

### 4.3. Future Application

Looking ahead, this project highlights the growing potential of large language models (LLMs) to enhance patient communication, particularly in head and neck cancer care. As tools such as GPT, Gemini, and Claude continue to advance, they could be harnessed to generate clear, personalised information that aligns with patients’ levels of understanding. With appropriate clinical oversight, AI-generated materials may help address patients’ concerns more effectively and support them in preparing consultations or understanding their diagnosis and treatment options. Integrating the reliability of professional health standards with the adaptability of AI systems for instance, through chatbot-based delivery could lead to more accurate and accessible patient resources. Future research should incorporate expert validation and patient feedback to ensure AI-generated content remains both clinically accurate and genuinely patient-centred, while establishing clear standards for the safe and responsible use of these technologies in clinical practice. Furthermore, the study used a single response per model, which may not capture the variability that can occur across multiple runs. Future research should examine how repeated prompts or multiple outputs from the same LLM affect consistency and content quality.

We also recommended that using standardized input fields or guided prompts could make patient–LLM interactions more structured, efficient, and clinically relevant. Additionally, response length may influence readability, as longer outputs often include more complex phrasing and technical details, leading to lower readability scores.

## 5. Conclusions

In our study, we found that LLMs (ChatGPT, Gemini, Claude) can produce patient information that is comparable to professional resources in terms of quality, understandability, actionability, and empathy. However, readability remains a key limitation, as these models often require specific prompting to generate content that aligns with recommended literacy standards for patient education. In practical terms, LLMs should be viewed as supplementary tools that can support, but not replace, professional medical and educational resources. Their integration into clinical communication should be guided by clinician oversight and tailored readability adjustments to ensure that information remains accurate, accessible, and safe for patient use.

To promote clearer and safer patient communication using LLMs, we recommend the following:

- Request plain-language output (e.g., “Write at a 6th-grade reading level”) when generating patient-facing responses.
- Apply a plain-language readability checklist before publication or dissemination.
- Involve healthcare professionals in reviewing AI-generated materials to ensure clinical accuracy and appropriateness.

## Figures and Tables

**Table 1:** Patient-Style Questions Developed for Each Quality-of-Life Domain.

Domains	Source	Questions
Pain	EORTC QLQ-H&N35/UW-QOL/FACT-H&N	“Will I have pain all the time? How can it be managed?”
Swallowing	EORTC QLQ-H&N35/UW-QOL/FACT-H&N	“Will I be able to chew or swallow normally again? Could I choke?”
Sense issues (taste/smell)	EORTC QLQ-H&N35	“Why does everything taste or smell different now? How can I cope with it?
Speech	EORTC QLQ-H&N35/UW-QOL/FACT-H&N	“Will my voice sound the same? Will people understand me?”
Social eating	EORTC QLQ-H&N35	“Is it going to be embarrassing to eat out with friends?”
Social contact	EORTC QLQ-H&N35/UW-QOL/FACT-H&N	“Will people treat me differently or avoid me because of this?”
Sexuality	EORTC QLQ-H&N35/FACT-H&N	“How might this affect my sex life or intimacy with my partner?”
Appearance	UW-QOL/FACT-H&N	“Will I look very different after treatment? Will I have scars?”
Activity	UW-QOL	“Will I still be able to do my normal daily activities?”
Recreation	UW-QOL	“Can I still enjoy hobbies or go out like before?”
Shoulder function	UW-QOL	“I’ve heard surgery can affect the shoulder—will I be able to move it normally?”
Saliva	UW-QOL	“Why is my mouth so dry or sticky? Will it always be like this?”
Mood and anxiety	UW-QOL/FACT-H&N	“I feel really down and anxious—is this normal, and what can help?”
Ability to eat desired foods	FACT-H&N	“Will I be able to eat my favourite foods again?”

**Table 2:** One-Way ANOVA Summary Table Comparing FRE Scores Across Information Sources.

Source of Variation	F	SS	MS	F crit
Between Groups		6716.1	2238.7	2.8
Within Groups		2557.9	49.2	
Total		9274.0		

SS: Sum of Squares, MS: Mean Square, F: F-ratio (or F-statistic), F crit: Critical value of F.

**Table 3:** Tukey’s Post Hoc Test for Pairwise Comparisons Between Information Sources.

Comparison	Mean Diff	95% CI Lower	95% CI Upper	*p*-Adj	Statistically Significant?
Claude vs. GPT	13.14	6.1	20.2	<0.001	Yes
Claude vs. Gemini	1.79	−5.3	8.8	0.907	No
Claude vs. Prof guideline	27.43	20.4	34.5	<0.001	Yes
GPT vs. Gemini	−11.36	−18.4	−4.3	0.0004	Yes
GPT vs. Prof guideline	14.29	7.3	21.3	<0.001	Yes
Gemini vs. Prof guideline	25.64	18.6	32.7	<0.001	Yes

**Table 4:** One-Way ANOVA Summary Table Comparing FKGL Scores Across Information Sources. F, df, *p*, η^2^/ω^2^).

Source of Variation	SS	MS	F crit
Between Groups	216.8	72.3	2.8
Within Groups	75.8	1.5	
Total	292.6		

SS: Sum of Squares, df: Degrees of Freedom, MS: Mean Square, F: F-ratio (or F-statistic), F crit: Critical value of F.

**Table 5:** Tukey’s Post Hoc Test for Pairwise Comparisons Between Information Sources.

Comparison	Mean Diff	95% CI Lower	95% CI Upper	*p*-Adj	Statistically Significant?
Claude vs. GPT	−3.12	−4.36	−1.88	<0.001	Yes
Claude vs. Gemini	−1.11	−2.35	+0.13	0.096	No
Claude vs. Prof guideline	−5.16	−6.40	−3.92	<0.001	Yes
GPT vs. Gemini	2.01	+0.77	+3.26	0.0004	Yes
GPT vs. Prof guideline	−2.04	−3.28	−0.79	0.0004	Yes
Gemini vs. Prof guideline	−4.05	−5.29	−2.81	<0.001	Yes

**Table 6:** One-Way ANOVA Summary Table Comparing DISCERN Scores Across Information Sources.

Source of Variation	SS	MS	F crit
Between Groups	14.63	4.88	2.78
Within Groups	528.21	10.16	
Total	542.84		

SS: Sum of Squares, MS: Mean Square, F crit: Critical value of F.

**Table 7:** One-Way ANOVA Summary Table Comparing PEMAT-Understandability Scores Across Information Sources.

Source of Variation	SS	MS	F crit
Between Groups	103.82	34.60	2.78
Within Groups	2646.42	50.89	
Total	2750.24		

SS: Sum of Squares, MS: Mean Square, F crit: Critical value of F.

**Table 8:** One-Way ANOVA Summary Table Comparing PEMAT-Actionability Scores Across Information Sources.

Source of Variation	SS	MS	F crit
Between Groups	199.77	66.59	2.78
Within Groups	4788.36	92.08	
Total	4988.13		

SS: Sum of Squares, MS: Mean Square, F crit: Critical value of F.

**Table 9:** One-Way ANOVA Summary Table Comparing ECCS Scores Across Information Sources.

Source of Variation	SS	MS	F crit
Between Groups	0.2	0.1	2.8
Within Groups	12.8	0.2	
Total	13.0		

SS: Sum of Squares, MS: Mean Square, F crit: Critical value of F.

## Data Availability

All data is available at: https://osf.io/q5d27 (accessed on 26 July 2025).

## Supplementary Materials

The following supporting information can be downloaded at: https://www.mdpi.com/article/10.3390/curroncol32120668/s1, File S1: STROBE checklist.

## Abbreviations

The following abbreviations are used in this manuscript:

LLMs	Large Language Models
AI	Artificial Intelligence
